# The Legislative Harvest

**Published:** 1901-06

**Authors:** 


### THE LEGISLATIVE HARVEST.

THE close of the sessions of most legislative bodies have been reached or are in their waning days, and the field may be surveyed as to the progress of legislation along veterinary lines.

In New York progress has been made in securing the better control of tuberculosis under more favorable auspices. The continued support of the State to one veterinary institution maintained ; the denial of like support to other equally worthy institutions, not without keen disappointment; the defeat of ulterior legislation in the interest of non-graduates achieved.

In Pennsylvania the defeat of legislation dangerous to the profession's advancement has been accomplished by an earnest, united protest of the profession. The continued support of the State Live-stock Sanitary Board is assured, and it is thought that increased assistance will be given it for more extended laboratory work.

In Michigan efforts to amend the present law and correct some of its serious defects have been fruitless in results, and continue to menace higher veterinary education and divide the profession.

In Montana most excellent results have been attained in laws regulating meat-inspection and milk-inspection, and these are under the most salutary provisions as to the qualifications essential for those charged with the enforcement of these laws.

In Indiana a law better defining the qualifications constituting a veterinary surgeon has been passed, and it is looked forward to as an aid in strengthening the profession.

In Missouri the effort to secure the passage of any law looking to defining the rights of practitioners of veterinary science has been defeated. The cause of defeat in this State was entirely due to the divided conditions of the profession and the rivalry of the veterinary colleges maintaining different curriculums.

